# Nutrient Digestibility, Growth, Mucosal Barrier Status, and Activity of Leucocytes From Head Kidney of Atlantic Salmon Fed Marine- or Plant-Derived Protein and Lipid Sources

**DOI:** 10.3389/fimmu.2020.623726

**Published:** 2021-02-19

**Authors:** Solveig L. Sørensen, Youngjin Park, Yangyang Gong, Ghana K. Vasanth, Dalia Dahle, Kjetil Korsnes, Tran Ha Phuong, Viswanath Kiron, Sjur Øyen, Karin Pittman, Mette Sørensen

**Affiliations:** ^1^ Faculty of Biosciences and Aquaculture, Nord University, Bodø, Norway; ^2^ Key Laboratory of East China Sea Fishery Resources Exploitation, Ministry of Agriculture, East China Sea Fisheries Research Institute, Chinese Academy of Fishery Sciences, Shanghai, China; ^3^ BioVivo Technologies AS, Bodø, Norway; ^4^ Department of Biosciences, University of Bergen, Bergen, Norway; ^5^ Quantidoc AS, Bergen, Norway

**Keywords:** Atlantic salmon, enteritis, mucosal barrier status, plant ingredients, mucin gene, antimicrobial genes, stereology, distal intestine

## Abstract

Nutrient digestibility, growth, and mucosal barrier status of fish skin, gills, and distal intestine were studied in Atlantic salmon fed feeds based on marine or plant-derived ingredients. The barrier status was assessed by considering the expression of four mucin genes, five genes that encode antimicrobial proteins, distal intestine micromorphology, and design-based stereology of the midgut epithelium. In addition, the head kidney leukocytes were examined using flow cytometry; to understand the differences in their counts and function. Five experimental feeds containing the main components i) fishmeal and fish oil (BG1), ii) soybean meal (BG2; to induce enteritis), iii) fishmeal as the main protein source and rapeseed oil as the main lipid source (BG3), iv) a mix of plant protein concentrates as the protein sources and fish oil as the lipid source (BG4), and v) plant and marine ingredients in the ratio 70:30 (BG5) were produced for the study. Atlantic salmon with initial weight 72.7 ± 1.2 g was offered the experimental feeds for 65 days. The results revealed that the weights of all fish groups doubled, except for fish fed BG2. Fish fed the BG2 diet had lower blood cholesterol concentration, developed enteritis, had lower expression of *muc2* in the distal intestine, and had a compromised barrier status in the intestine. Expression of both the mucin genes and genes that encode antimicrobial peptides were tissue-specific and some were significantly affected by diet. The fish fed BG1 and BG3 had more head kidney lymphocyte-like cells compared to BG5-fed fish, and the phagocytic activity of macrophage-like cells from the head kidney was the highest in fish fed BG1. The intestinal micromorphology and the mucosal mapping suggest two different ways by which plant-based diets can alter the gut barrier status; by either reducing the mucous cell sizes, volumetric densities and barrier status (as noted for BG2) or increasing volumetric density of mucous cells (as observed for BG4 and BG5). The results of the compromised intestinal barrier in fish fed plant ingredients should be further confirmed through transcriptomic and immunohistochemical studies to refine ingredient composition for sustainable and acceptable healthy diets.

## Introduction

Mucosal surfaces of fishes, the skin, gills, and gastrointestinal tract, are important barriers that protect the host from pathogens and infections. The barriers include a mucosal epithelium which is covered by mucus and a wide range of components such as antimicrobial peptides that inhibit the entry of pathogens (1, 2). Mucus contains O-glycosylated proteins called mucins, and the expression of mucin genes in fish is altered by parasite infection (3) and fish density- and handling-related stress (4). The mucin glycosylation itself plays a key role in disease resistance in fish (5) and is affected both by the origin and size of Atlantic salmon (6). Antimicrobial peptides (AMPs) are also important components of the innate immune system in fish (2). The AMPs are classified into different families which show broad-spectrum antimicrobial activity to overcome the different resistance mechanisms activated by microbial organisms (2, 7, 8). The innate immune system plays a key role in keeping fish healthy in intensive aquaculture systems, especially the components at the semipermeable mucosal epithelia in the gut (9–11). Dietary interventions are known to strengthen the intestinal barrier in mice and humans, thereby allowing the organ to carry out its intended functions (12). However, little information is available as to how the intensive production systems and use of modern diets affect the gut barrier function of fishes.

Modern diets are formulated on the presumption that fish do not have a need for specific ingredients, but combinations of different ingredients can help meet the nutrient requirements of the farmed species. Fishmeal (FM) and fish oil (FO) are still considered to be the gold standard feed ingredients. However, their use in commercial fish feeds is reduced to a minimum because of static supply, increasing demand resulting in increasing prices and debates about sustainability when fish is used to feed fish. Commercial feeds used in Norwegian salmon farming are based on plant-derived products, which constitute 71% of the feed ingredients, while the marine feed ingredients is reduced to approximately ~25% (13). Soy protein concentrate has become the key protein source and rapeseed oil the primary oil source in present-day salmon feeds (13, 14). However, these ingredients have certain drawbacks. Feeding rapeseed oil is known to affect the n-3/n-6 ratio in the fillets of farmed salmon. Use of plant products with unfavourable n-6/n-3 ratio or diets without eicosapentaenoic acid (EPA) may bring about histomorphological changes in the intestine and can reduce fish growth (15, 16). Many studies have shown that the intestinal structure, microbiota and ion and water transport of Atlantic salmon are affected by the feed ingredients (17–19). However, further research is needed to understand the effect of feed ingredients on the immune defense of the fish, especially at the intestinal level.

Most studies have employed fishmeal-based diets to evaluate the impact of plant ingredients on salmon; the researchers have replaced either fishmeal with plant protein or fish oil with plant oil. Few studies have investigated the effect of different combinations of protein and oil derived from marine and plant origin on the growth and health of the fish. The aim of this study was to investigate the combined effect of replacing marine proteins and lipids with a mixture of plant-derived protein concentrates and oil on growth, nutrient digestibility, mucosal barrier status and systemic immune responses. The barrier status was assessed based on the expression of mucin genes in the skin, gills, and distal intestine, the expression of genes that encode antimicrobial proteins in the skin and distal intestine, histological changes in the distal intestine and information from design-based stereology of the midgut epithelium. Stereology was used to evaluate the mucosal barrier function because this type of mucosal mapping is more sensitive and independent of section orientation (11, 20). Furthermore, to understand the systemic effect, head kidney leukocytes were examined using flow cytometry.

## Material and Methods

### Experimental Design and Feeds

The study used five experimental diets: a control diet (BG1) based on fishmeal and fish oil; a diet containing 20% soybean meal and 30% fishmeal and fish oil (BG2); a diet with fishmeal and rapeseed oil (BG3); a diet based on a mix of plant protein concentrates as the main protein source (soy protein concentrate, pea protein concentrate and corn gluten meal) and fish oil (BG4); and one diet resembling a commercial diet with the same protein ingredients as in BG4 and a mix of rapeseed oil and fish oil (BG5; **Table 1**). All diets were supplemented with crystalline amino acids (lysine, histidine, methionine and threonine) and inorganic phosphate (**Table 1** and **Supplementary Table 1**). Diets also contained 0.01% yttrium oxide as an inert marker for digestibility measurements.

**Table 1:** Ingredient composition (%) of the experimental feeds.

	BG1	BG2	BG3	BG4	BG5
Fishmeal	50	30	50	10	10
Wheat meal	13.85	6.55	13.85	6.05	6.05
Wheat gluten	5	10	5	10	10
Soy protein concentrate	0	0	0	20	20
Soybean meal	0	20	0	0	0
Corn Gluten	0	0	0	9	9
Pea protein concentrate	0	0	0	9	9
Fish oil	25	26.4	3.8	27.5	7.7
Rapeseed oil	0	0	21.2	0	19.8
Mineral premix	0.59	0.59	0.59	0.59	0.59
Vitamin premix	2	2	2	2	2
Monosodium Phosphate	2.5	2.5	2.5	2.5	2.5
Carop. Pink (10% Astax)	0.05	0.05	0.05	0.05	0.05
Yttrium oxide	0.01	0.01	0.01	0.01	0.01
Choline	0.5	0.5	0.5	0.5	0.5
Methionine	0.3	0.6	0.3	0.9	0.9
Lysine	0	0.5	0	1.2	1.2
Threonine	0	0.1	0	0.4	0.4
Histidine	0.2	0.2	0.2	0.3	0.3

The five feed mixes were prepared and homogenized (30 min) using a horizontal ribbon mixer. The feed mixes were conditioned with steam and water in an atmospheric double differential preconditioner (DDC) prior to extrusion in a TX-52 co-rotating, fully intermeshing twin-screw extruder (Wenger Manufacturing Inc., Sabetha, KS, USA). The temperature of the feed mash entering the extruder was 86–88°C. Temperature at the extruder outlet were 120°C for BG1 and BG3, 128°C for BG2 and 137°C for BG4 and BG5. Three of the diets, BG2, BG4, and BG5 had lower wheat content in the recipe, and hence more moisture was added as heat into the DDC to ensure expansion. The extruder outlet had 24 circular 2.5 mm die holes. The wet extrudates were cut at the die surface with a rotating knife. To ensure the pellet quality, pellet samples were visually inspected after achievement of steady state conditions in the preconditioner and extruder. The extrudate was dried in a hot air dual layer carousel dryer (Paul Klockner, Nistertal, Germany) at a constant air temperature (77°C) to obtain final products of approximately 7–8% moisture. Then each of the diets were coated with oil in an experimental vacuum coater (Pegasus PG-10VC LAB, Dinnissen B.V., Netherlands). Immediately after coating, diets were packed in sealed plastic buckets and shipped to the research site.

### Fish and Feeding

Atlantic salmon (*Salmo salar*) post-smolts were obtained from Cermaq, Hopen, Bodø, Norway (Aquagen strain, Aquagen AS, Trondheim, Norway) and maintained at the Research Station, Nord University, Bodø, Norway. At the start of the experiment, a total of 1100 fish (initial weight 72.7 ± 1.4 g) (mean ± SD) were randomly allocated to 20 experimental units (n = 4 tanks per treatment group).

The feeding experiment was carried out in a flow-through system. In total, 20 circular fiberglass tanks (1100 L) were used for the study. Each tank was supplied with water pumped from a depth of 250 m from Saltenfjorden. During the experiment, water flow rate was maintained at 1000 L per hour, and the average temperature and salinity of the rearing water were 7.6°C and 35‰, respectively. Oxygen saturation was always above 85% measured at the water outlet. A 24-h photoperiod was maintained throughout the experimental period. The fish were fed *ad libitum* using automatic feeders (Arvo Tech, Finland) for 12 h per day from 08:00–20:00 (divided into eight feedings: 08:00–10:00, 10:00–12:00, 12:00–14:00, 14:00–16:00, 16:00–18:00, 18:00–19:00, and 19:00–20:00) during the 65-day feeding trial.

### Fish Sampling and Data Collection

At the beginning and end of the experiment, all fish (1100) were individually weighed, and their total lengths recorded. Before handling, fish were anesthetized using tricainemethanesulfonate (MS 222, 140 mg/L). Feces for digestibility determination was obtained by stripping individual fish. Feces from all individuals from a tank were pooled into one sample to obtain a value from a particular tank. The fecal samples that were immediately transferred to -20°C were used for further analyses.

For the histology and design-based stereology studies, distal intestine and mid intestine samples, respectively were collected as described in our previous publications (20–24). In addition, skin, gill and distal intestine samples were obtained for the gene expression analysis, and our standard protocols (21–23) were used in the present study also. For the cell study, the head kidney (HK) was collected at the end of experiment. These tissues were immediately transferred to 15 ml tubes to make a total volume of 4 ml in ice-cold Leibovitz’s L-15 Medium (L-15; Sigma-Aldrich, Oslo, Norway), supplemented with 100 µg/ml gentamicin sulphate (Sigma), 2 mM L-glutamine (Sigma), and 15mM HEPES (Sigma).

### Biochemical and Cholesterol Analyses

Frozen fecal samples were freeze dried (VirTis benchtop, U.S.A.) for 72 h at -76°C and at a pressure of 20 bar. The moisture, protein, lipid, ash, energy and yttrium contents of the feed and freeze-dried feces were determined as described in Sørensen et al. (22). Blood was drawn from the caudal vein of 12 fish/feed, into lithium heparin vacutainers and immediately spun at 703.2 x *g* for 10 min at 4°C. Cholesterol level in the plasma was measured by application of 115 µl plasma to a T4/Cholesterol rotor cassette (Profile #500-0037, Abaxis, CA, US), and analyzed by a VETSCAN Chemistry Analyzer (VETSCAN VS2, Abaxis, CA, US). Cholesterol was only analyzed in fish from BG1-BG4 due to lack of cassettes to analyze fish from BG5.

### Mucosal Mapping

Samples for mucosal mapping with design-based stereology were collected at the end of the feeding experiment (day 65). Approximately 2 cm of the anterior part of the mid intestine from four fish (three tanks per diet group) were collected for this study—in total 12 samples per diet group. Luminal contents were first rinsed out with 10% neutral buffered formalin, and then the tissues were fixed in 10% formalin for 48 h. The fixed samples were dehydrated in an alcohol gradient, equilibrated in xylene and embedded in paraffin blocks. Approximately, 5 μm thick longitudinal sections were cut using a microtome and mounted onto a glass slide. The sections were stained with Alcian Blue pH 2.5—Periodic Acid Schiff’s reagent (25) and mounted with Pertex medium.

All slides were scanned in batches using a Hamamatsu NanoZoomer S60 with a source lens; at 40x magnification and saved as high-resolution digital images in NDPI-format. The digital files were examined using NDP.view 2.6.8 (Free edition, Hamamatsu Photonics K.K. 2016). Mucosal mapping of the digitized slides was performed using the MucoMaster2 (Quantidoc AS, 2019) software according to Pittman et al. (26, 27). Blinded stereological analysis was done, maintaining the anonymity of the diet groups until the completion of the analysis. Regions of interest were manually drawn over the mucosal folds and lamina propria of each fish midgut. An unbiased selection of about 100 mucosal cells was performed to carry out the measurements for each slide as described in Pittman et al. (26) Epithelial area and mucous cell area were measured using stereological probes, followed by counting of mucous cells. Mean area of the mucous cells and percentage of epithelial with mucous cells were exported to Microsoft Excel for Office 20 365 MSO version 1908 (Microsoft Corporation, 2019). The barrier status as described in Dang et al. (20) was calculated using the mean mucous area, mucous number and epithelial area.

### Distal Intestinal Micromorphology

Sections of the distal intestine were prepared as described under *Mucosal Mapping*. Slides were examined using microscope Olympus BX51 at 100x total magnification and photomicrographs were captured employing Camera SC180 (Olympus Europa GmbH, Hamburg, Germany) and processed using the imaging software CellEntry (Soft Imaging System GmbH, Munster, Germany).

### Gene Expression Analysis

Tissues for gene expression analysis were sampled from the second gill arch (left side of the fish), skin (below dorsal fin), and distal intestine of 16 fish per diet group (four fish per tank). These tissues were immediately placed in tubes filled with RNA later^®^ (Ambion Inc., Austin, Texas, United States), and stored at -20°C until further analysis.

The relative mRNA levels of mucin genes (*muc2*, *muc5ac1*, *muc5ac2*, and *muc5b*) in the distal intestine, skin and gills and antimicrobial protein genes (*defensin 1 - def1; defensin 2 - def2, defensin 3 - def3, defensin 4 - def4;cathelicidin 1 - cathl1*) in the distal intestine and skin were examined in this study. The primer sequences for all target and reference genes are presented in **Supplementary Table 2**. Primers were purchased from Eurofins Genomics (Luxembourg, Luxembourg).

RNA was extracted from the samples using E-Z 96 Total RNA Kit (Omega Bio-Tek, USA). Roughly 100 mg of the tissue sample was removed from RNA later^®^ and homogenized using Zirconium oxide beads (1.4 mm; Percellys, Tarnos, France) and TRK lysis buffer in a capped free standing tube (VWR International, Oslo, Norway) at 6000 rpm. The resulting mixture was centrifuged (18,000 × g, 20°C) to obtain a clear supernatant. Briefly, 300 µl supernatant was added to 300 µl of 70% ethanol and mixed, before this mixture was added to the E-Z 96 RNA plate which contains an RNA HiBind^®^ matrix in each well. Centrifugation (3000 rpm, 15 min) was used to draw the sample through the well, followed by several steps of buffer washes according to the kit instructions. Finally, the purified RNA was obtained by adding 65–75 μl of RNase-free water (5 Prime GmbH, Hilden, Germany) to each well and a final centrifugation.

Extracted RNA was quantified by Qubit™ RNA broad-range assay kit (Life Technologies, Carlsbad, USA) on a Qubit 3.0 Fluorometer (Life Technologies, Carlsbad, USA) and diluted with RNase-free water if necessary. cDNA synthesis was done with QuantiTect™ Reverse Transcription Kit (Quiagen GmbH, Hilden, Germany) employing 1000 ng of RNA and a reaction volume of 20 µl per sample, according to the manufacturer’s instructions. The cDNA samples were diluted with nuclease free water by a factor of 10 before continuing with qPCR.

The qPCR was performed on a LightCycler^®^ 96 (Roche Life Science) using Fast SYBR^®^ Green Real-Time PCR Master Mix (Applied Biosystems, Carlsbad, USA). Each reaction contained 5 μl of Fast SYBR^®^ Green PCR Master Mix, 1 µl primer mix (200 nM), and 4 µl cDNA (0.5 ng/µl). Reactions (n = 16 per diet) were performed in duplicate. Thermal cycling conditions were: initial holding at 95°C for 20 s, 40 cycles of denaturation at 95°C (3 s), and annealing/extension at 60°C (30 s).

A standard curve with known concentrations was prepared for each primer in order to calculate the gene expression. This was done by pooling RNA from every sample, reverse transcribing the pooled RNA as described above, and using the resulting cDNA to create a 6-point threefold dilution series. The equation E = (10^(−1/m)− 1)*100 was used to calculate the efficiency of the primers; E, efficiency and m, slope of the standard curve (**Supplementary Table 2**). Using geNorm (28) a normalization factor was computed for each sample based on the relative quantities of the two most stable genes from among the set of four reference genes, namely elongation factor 1AB (*ef1ab*), ribosomal protein L13 (*rpl13*), ribosomal protein S29 (*rps29*), and ubiquitin (*ubi*). The expression levels of all the target genes were calculated relative to the normalization factor.

### Head Kidney Leucocytes

Head kidney (HK) cells (six fish/group) were harvested employing the protocols described for Atlantic salmon HK cells (29). The leucocyte fraction was employed for analysis of the lymphocyte counts. The monocyte/macrophage fraction was allowed to adhere on a petri dish for 3 days at 12°C. The adherent cells were detached by washing three times with 1.5 ml ice-cold phosphate-buffered saline (PBS) supplemented with 5mM EDTA (Sigma), and centrifuged at 500 × g for 5 min at 4°C. The cells were counted using a portable cell counter (Scepter™ 2.0 cell counter, EMD Millipore, Darmstadt, Germany). The flow cytometric analyses were performed as described by Park et al. (29), employing ImageStream^®^X Mk II Imaging Flow Cytometer (Luminex Corporation, Austin, TX, USA). Cell analyses were performed on 20,000 cells; lymphocyte-like cell population was determined based on the positivity of cells to salmon IgM while other cell populations (monocyte/macrophages) were identified based on morphological characteristics (29). Phagocytosis was studied using fluorescent bio-particles designed for flow cytometry, as detailed in our previous publication (29). Phagocytic ability and phagocytic capacity are presented to indicate phagocytosis; the former parameter is the percent of phagocytic cells, and the latter one is calculated as the mean number of particles per phagocytic cell.

### Calculations and Statistical Analysis

Fish growth performance was analyzed using the following equations.

$$\text{Weight gain}(\%)=\left(\frac{W_{f}-W_{i}}{W_{i}}\right)\times 100$$

Where Wf = final body weight of fish (g/fish) and Wi = initial body weight of fish (g/fish)

$$\text{Specific Growth Rate}\;\left(\%\;day^{-1}\right)=\left(\frac{Ln\;\left(W_{f}\right)-\text{Ln}\;\left(W_{i}\right)}{\text{No.of feeding days}}\right)\times 100$$

$$\mathrm{Thermal growth coefficient}\;(\text{TGC})=\frac{{\left(W_{f}\right)}^{1/3}-{\left(W_{i}\right)}^{1/3}\;}{\left(\text{T}\times \text{d}\right)}\times 1000$$

where T is the temperature in °C and d is time in days.

Apparent Digestibility Coefficient (ADC) of nutrients and dry matter were calculated according to following equations:

$$ADC_{nutrient\;}=\left[1-\left(\frac{Marker_{feed}\times Nutrient_{feces}}{Marker_{feces}\times Nutritent_{feed}}\right)\right]\times 100$$

$$ADC{\;}_{dry\;matter}=\left[1-\left(\frac{Marker_{feed}}{Marker_{feces}}\right)\right]\times 100$$

where *Marker_feed_* and *Marker_feces_* represent the marker content (% dry matter) of the feed and feces, respectively, and *Nutrient_feed_*and *Nutrient_feces_* represent the nutrient contents (% dry matter) in the feed and feces, respectively. Tank was used as the experimental unit.

The mucous cell-based barrier status was calculated using the following formula:

[1Mucous cell area[Mucous cell area x mucous numberEpithelial area×100]]×1000

Statistical analyses were performed using SPSS 22.0 software and R packages for Windows. The data were tested for normality (Shapiro–Wilk normality test) and equality of variance (Levene’s test). For parametric data, one way analysis of variance (ANOVA) was performed after checking for equal variance. Tukey’s multiple comparison test was used to identify the significant differences among the means of the dietary groups. For non-parametric data, Kruskal-Wallis test, followed by Dunn’s multiple comparison test, was performed to decipher the significant differences between the groups. A significance level of p < 0.05 was chosen to indicate the differences.

## Results

### Apparent Digestibility Coefficients

The dry matter content in feces was significantly higher in BG1- and BG3-fed fish (14%–15%) compared with BG2-, BG4-, and BG5-fed fish (10%–11%). We observed significant differences for the digestibility values of dry matter (DM), protein, lipid, ash and energy of the five feeds (**Table 2**). The DM digestibility was significantly lower in BG4-fed (59%) fish compared to BG2 (66%) and BG3 (68%), while no differences were noted among fish fed BG1, BG2, BG3, and BG5. Protein digestibility was lowest (significantly) in fish fed the BG1 (81%) compared to the other groups (85%–88%). Lipid digestibility was the highest in fish fed BG3 (96%) and BG5 (95%), and the lowest in fish fed BG2 (87%). Digestibility value of ash in BG2-fed fish was positive (1%), while those of fish fed other diets were negative (9%–33%). Energy digestibility was significantly higher in fish fed the BG3 (84%) compared to the other groups (73%–78%).

**Table 2:** Dry matter content in feces and apparent digestibility coefficients (ADC %) of dry matter (DM), lipid, protein, ash, and energy in Atlantic salmon fed the experimental diets.

	BG1	BG2	BG3	BG4	BG5	p value
DM	14.5 ± 0.5^a^	10.4 ± 0.4^b^	13.8 ± 0.8^a^	11.2 ± 0.4^b^	11.4 ± 0.4^b^	<0.001
ADC %						
DM	62.1 ± 3.1^ab^	66.1 ± 0.6^a^	68.4 ± 1.2^a^	59.0 ± 3.4^b^	63.6 ± 5.4^ab^	0.007
Protein	81.3 ± 1.7^b^	86.1 ± 0.3^a^	85.5 ± 0.6^a^	86.6 ± 1.4^a^	88.1 ± 2.2^a^	<0.001
Lipid	90.6 ± 1.3^b^	87.4 ± 0.2^c^	96.4 ± 0.2^a^	92.0 ± 0.9^b^	95.4 ± 2.4^a^	<0.001
Ash	-14.1 ± 10.7^ab^	0.9 ± 4.6^a^	-8.6 ± 1.8^a^	-33.2 ± 10.6^b^	-21.0 ± 17.5^ab^	0.005
Energy	77.6 ± 1.7^b^	77.3 ± 0.6^b^	83.8 ± 0.8^a^	73.1 ± 2.4^b^	77.0 ± 3.6^b^	<0.001

BG1: Fishmeal + Fish oil diet; BG2: Soybean meal diet; BG3: Fishmeal + Plant oil diet; BG4: Plant ingredients + Fish oil diet; BG5: Plant ingredients + Plant oil diet.

Values are expressed as mean ± SD (n=4 replicates). Values in the same row with different superscript letters indicate significant differences (p <.05).

### Growth Performance

The weight gain and growth rate are given in **Table 3**. The fish grew from an initial average weight of 70 g to a final average body weight of 150 g during the experimental period of 65 days. Significantly lower final body weight (138 g), weight gain (94%), thermal growth coefficient (2.1) was noted in fish fed the BG2 compared to the fish fed BG3 (158 g, 117%, 2.5, respectively). No differences in final body weight, weight gain, specific growth rate and thermal growth coefficient were noted for fish belonging to the different dietary treatments. Five fish died during the experiment, but mortality was not related to feed groups.

**Table 3:** Growth performance of Atlantic salmon for the experimental period.

	BG1	BG2	BG3	BG4	BG5	p value
IBW	72.4 ± 1.2	71.3 ± 1.0	72.9 ± 1.7	73.5 ± 1.4	73.5 ± 0.9	0.15
FBW	152.3 ± 4.5^a^	138.3 ± 5.3^b^	158.4 ± 5.9^a^	150.7 ± 9.4^ab^	150.3 ± 4.9^ab^	0.01
WG	110.2 ± 7.9^ab^	93.8 ± 7.0^b^	117.2 ± 3.3^a^	105.1 ± 16.3^ab^	104.7 ± 8.2^ab^	0.04
SGR	1.1 ± 0.1	1.0 ± 0.1	1.2 ± 0.1	1.1 ± 0.1	1.0 ± 0.1	0.11
TGC	2.4 ± 0.1^ab^	2.1 ± 0.1^b^	2.5 ± 0.1^a^	2.3 ± 0.3^ab^	2.3 ± 0.1^ab^	0.05

BG1: Fishmeal + Fish oil diet; BG2: Soybean meal diet; BG3: Fishmeal + Plant oil diet; BG4: Plant ingredients + Fish oil diet; BG5: Plant ingredients + Plant oil diet.

IBW, Initial body weight (g); FBW, Final body weight (g); WG, Weight gain (%); SGR, Specific growth rate (% day^-1^); TGC, Thermal growth coefficient.

Values are expressed as mean ± SD (n=4 replicates). Values in the same row with different superscript letters show significant differences (p <.05).

### Cholesterol

**Figure 1:**
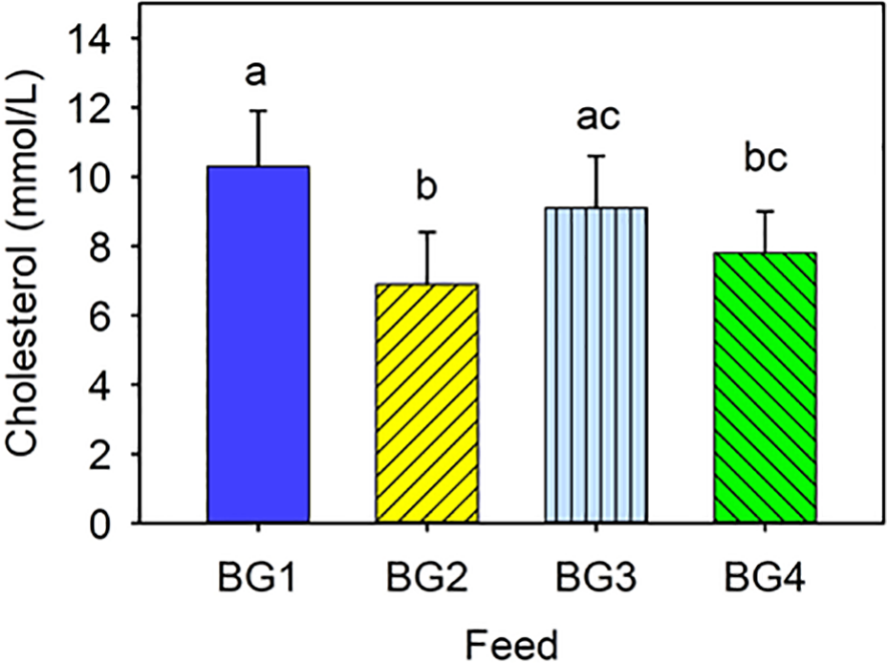
Cholesterol level in the blood of Atlantic salmon fed different experimental diets. BG1: Fishmeal + Fish oil diet; BG2: Soybean meal diet; BG3: Fishmeal + Plant oil diet; BG4: Plant ingredients + Fish oil diet. Values are expressed as mean ± SD (n=12 fish per diet group). Different letters above the bars indicate significant differences (p < .05).

Cholesterol concentration in blood ranged from 7 to 10 Mmol/L, and certain values were significantly differences (**Figure 1**). Cholesterol level was the highest in fish fed fishmeal-based diets, BG1 and BG3, and the lowest in those fed BG2. Fish fed the BG4 had lower cholesterol than those fed BG1, but not significantly different from BG3-fed fish.

### Histology of the Distal Intestine

**Figure 2:**
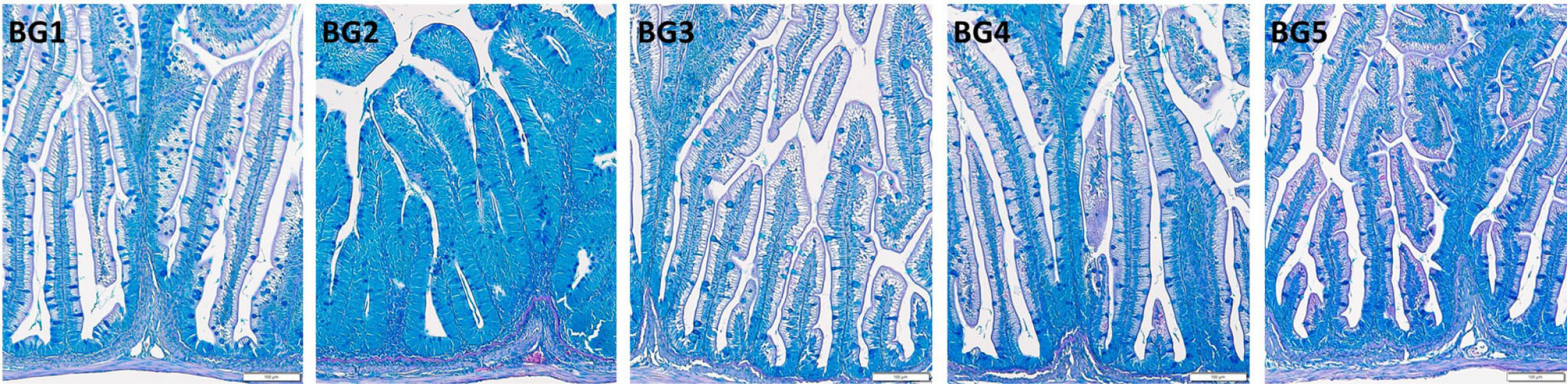
Histomorphology of the distal intestine of Atlantic salmon fed diets BG1-5. BG1: Fishmeal + Fish oil diet; BG2: Soybean meal diet; BG3: Fishmeal + Plant oil diet; BG4: Plant ingredients + Fish oil diet; BG5: Plant ingredients + Plant oil diet.

Micromorphology of the distal intestine samples is shown in **Figure 2**. Inflammatory response in BG2-fed group was evident from the aberrant lamina propria, widened villi, villi fusion and infiltration of inflammatory cells into lamina propria from base of intestinal mucosa. In addition, nuclei of intestinal absorptive cells were displaced and supranuclear vacuoles were also absent in the distal intestine of BG2-fed fish.

Fish fed the BG1 and BG3 had distal intestine with normal features. Enterocytes had a columnar shape, with nuclei situated near the lamina propria. Supranuclear vacuoles were present and the tissue had a normal distribution of goblet cells. Lamina propria had a slender and delicate core, and normal intraepithelial leucocyte infiltration was observed in BG1- and BG3-fed fish. Fish fed BG4 and BG5 also had normally positioned cell nuclei, and the typical distribution of goblet cells. However, the supranuclear vacuoles were smaller in size compared to BG1.

### Mucosal Mapping

**Figure 3:**
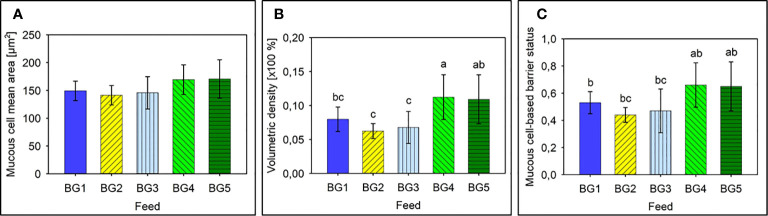
Mucus cell-based analysis to assess the barrier status in the mid intestine of Atlantic salmon. **(A)** Mean area of mucus cells present in the mid intestinal epithelium of Atlantic salmon. **(B)** Mean volumetric density of mucous cells present in the mid intestine of Atlantic salmon. **(C)** Barrier status of the mid intestine of Atlantic salmon. BG1: Fishmeal + Fish oil diet BG1: Fishmeal + Fish oil diet; BG2: Soybean meal diet; BG3: Fishmeal + Plant oil diet; BG4: Plant ingredients + Fish oil diet; BG5: Plant ingredients + Plant oil diet. Values are expressed as mean ± SD (n=12 fish per diet group). Different letters above the bars indicate significant differences among diet groups.

The mean area of intestinal mucous cells for the 60 fish sampled was around 155.3 ± 3,6 μm^2^, for the five diet groups. The mucous cells’ mean area per diet group was not significantly different (**Figure 3A**).

Average intestinal mucous cell density ranged from about 6% to about 11% and density of the mucous cells differed among diet groups (p < 0.05; **Figure 3B**). Fish fed BG2 and BG3 had mucous cell volumetric densities that was significantly lower than fish fed diets BG4 and BG5 (p < 0.001). Interestingly the marine diet BG1 also had a volumetric density of mucous cells in the epithelium that was significantly lower than BG4 (p < 0.05), and the values indicated a strong tendency towards a lower volumetric density than fish fed BG5 (p = 0.057).

The mucous cell-based barrier status values of the different fish groups also indicated a strong tendency to differ (p = 0.062). Fish fed BG2 had the lowest average barrier status (0.440 ± 0.055) and those fed BG1, BG2 and BG3 had a significantly lower barrier status than fish fed diets BG4 and BG5 (**Figure 3C**, p < 0.01).

### Expression of Mucin Genes and Antimicrobial Protein-Encoding Genes

**Figure 4:**
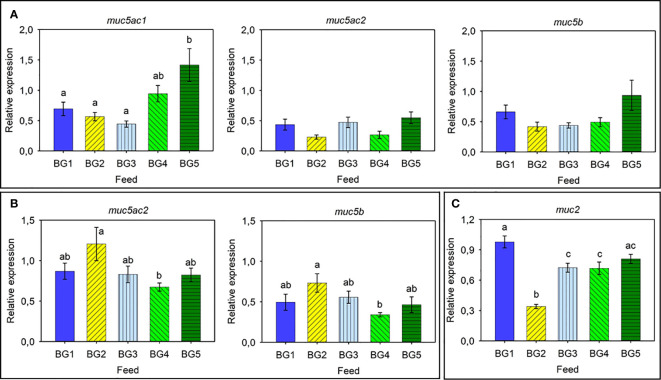
Relative expression of mucin-related genes in Atlantic salmon. **(A)** Skin: *muc5ac1*, *muc5ac2*, and *muc5b*. **(B)** Gills: *muc5ac2* and *muc5b*. **(C)** Distal intestine: *muc2*. BG1: Fishmeal + Fish oil diet; BG2: Soybean meal diet; BG3: Fishmeal + Plant oil diet; BG4: Plant ingredients + Fish oil diet; BG5: Plant ingredients + Plant oil diet. Values are expressed as mean ± SD (n=12 fish per diet group). Different letters above the bars indicate significant differences (p < .05). Expression of *muc2* was too low to be quantified in the skin. Expression of *muc5ac1* and *muc2* was too low to be quantified in the gills. Expression of *muc5ac1*, *muc5ac2*, and *muc5b* was too low to be quantified in the distal intestine.

The relative expression of mucin genes in Atlantic salmon skin, gills, and distal intestine is shown in **Figure 4**, respectively. Expression of all four mucin genes were analyzed for all three tissues, and the expressional pattern was found to be tissue-specific.

The skin expressed *muc5ac1, muc5ac2*, and *muc5b* (**Figure 4A**). The expression of *muc5ac1* was relatively higher than those of the other two genes, and significant differences were observed only for the *muc5ac1* gene. The fish fed BG5 diet had the highest relative expression of the *muc5ac1* gene; approximately 3-fold higher compared to other groups. On the other hand, fish fed BG4 tended to have higher expression (2-fold) than those fed BG1-BG3 but lower (-1.5-fold) than BG5-fed fish.

The gills expressed the two genes *muc5ac2* and *muc5b*, and these genes showed an overall higher relative expression (**Figure 4B**). A similar relative expression pattern was noted for both the genes; the highest value (2.1-fold) for fish fed BG2 and lowest in fish fed BG4.

The distal intestine expressed *muc2* (**Figure 4C**). Fish fed BG2 had a significantly reduced (-3.2-fold compared to BG1) expression compared to all the other fish groups. The BG1 group had the highest relative expression (1.3-fold) and was significantly different compared to BG2, BG3 and BG4.

**Figure 5:**
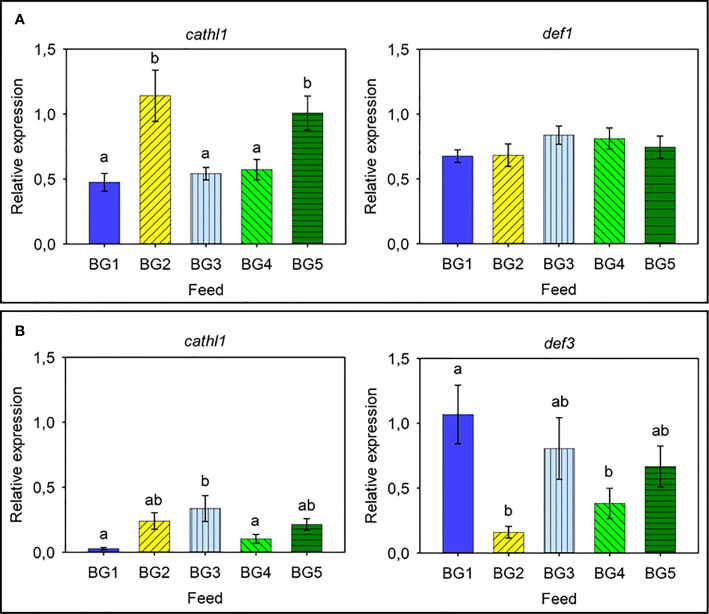
Relative expression of antimicrobial protein genes in the skin and distal intestine of Atlantic salmon. **(A)** Skin *cathl1* and *def1*. **(B)** Distal intestine *cathl1* and *def3*. BG1: Fishmeal + Fish oil diet; BG2: Soybean meal diet; BG3: Fishmeal + Plant oil diet; BG4: Plant ingredients + Fish oil diet; BG5: Plant ingredients + Plant oil diet. Values are expressed as mean ± SD (n=12 fish per diet group). Different letters indicate significant differences (p < .05). Expression of *def2*, *def3*, and *def4* was too low to be quantified in the skin. Likewise, the expression of *def1*, *def2*, and *def4* was too low to be quantified in the distal intestine.

As for the relative expression of AMPs in Atlantic salmon skin (**Figure 5A**) and distal intestine (**Figure 5B**), the relative expression of *cathl1* and *def1* in the skin of Atlantic salmon was relatively high and the expression of *cathl1* was significantly higher in fish fed BG2 (2.5-fold compared to BG1 and BG3-4) and BG5 (2-fold, **Figure 5A**). We did not observe any differences in the expression of *def1* in the different fish groups. In the distal intestine, *def3* had higher relative expression than *cathl1* (**Figure 5B**). Expression of both genes in the diet groups differed significantly. The expression of *cathl1* was significantly higher in fish fed BG3 compared to those fed BG1 and BG4. The *def3* had the highest expression (3.7-fold) in fish fed BG1 and lowest for those fed BG2 and BG4.

### Salmon Head Kidney Lymphocyte-Like Cell Population and Phagocytic Activity of Macrophage-Like Cells

**Figure 6:**
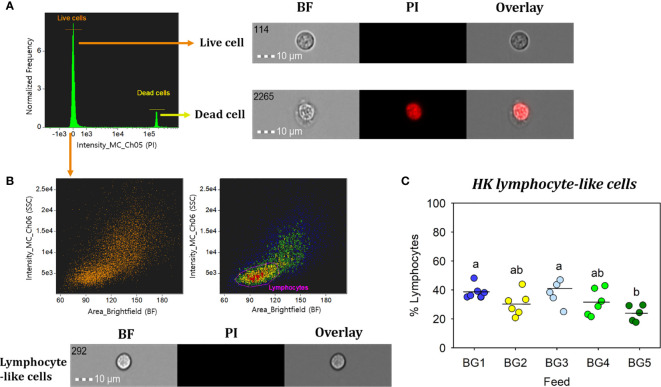
Percentage of head kidney lymphocyte-like cells from Atlantic salmon fed different experimental diets. BG1: Fishmeal + Fish oil diet; BG2: Soybean meal diet; BG3: Fishmeal + Plant oil diet; BG4: Plant ingredients + Fish oil diet; BG5: Plant ingredients + Plant oil diet. **(A)** Live cells (orange) were separated by excluding the dead cells (yellow); by staining with propidium iodide (PI). **(B)** Brightfield (BF) area (cell size) vs. side scatter (SSC) intensity (cell internal complexity) plot showing the HK leucocyte population. **(C)** Percentage of HK lymphocyte-like cells from fish (n=6) fed different experimental diets. Statistically significant differences (*p* < 0.05) between dietary groups are indicated by different letters. All cell images were captured with 40× objective. Scale bar = 10 µm. BF, brightfield; PI, propidium iodide.

The percentages of lymphocyte-like cells in the head kidney from fish fed BG1 (39%) and BG3 (41%) were significantly higher than that of fish fed BG5 (24%; **Figure 6**; p < 0.05). However, there was no significant difference between the counts of fish fed BG1 and BG3 (41%; p > 0.05) or those fed BG2 (30%) and BG4 (32%).

**Figure 7:**
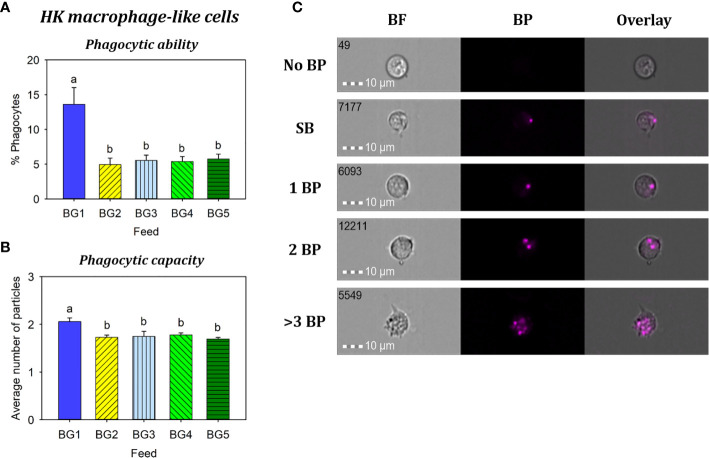
Phagocytosis of head kidney macrophage-like cells from Atlantic salmon fed different experimental diets. BG1: Fishmeal + Fish oil diet; BG2: Soybean meal diet; BG3: Fishmeal + Plant oil diet; BG4: Plant ingredients + Fish oil diet; BG5: Plant ingredients + Plant oil diet. **(A)** Percent of phagocytic cells. **(B)** mean number of particles ingested per phagocytic cell. **(C)** Representative cell images indicate cells with no BP, SB, and 1BP, 2BP, and >3BP. Statistically significant differences (*p* < 0.05) between dietary groups are indicated by different letters. Bar plots show mean ± SD, n = 6. All cell images were captured with 40× objective. Scale bar = 10 µm. SB, surface-binding particles; 1 BP, 2 BP, and > 3 BP, one to three internalized bio-particles; BF, brightfield.

Phagocytic ability (**Figure 7A**) and capacity (**Figures 7B, C**) of HK macrophage-like cells from fish fed BG1 were significantly higher than those fed the other diets (p < 0.001). There were no significant differences among the fish fed BG2-5 (p > 0.05).

## Discussion

The experimental diets were formulated to investigate nutrient digestibility, growth, mucosal barrier status, and activity of leucocytes from head kidney of the fish fed marine- or plant-derived protein and lipid sources. Plant protein concentrates were chosen to evade the negative effect of carbohydrate and antinutritional factors in plant ingredients on fish health, as noted by other researchers (30–33). Furthermore, feeding studies with Atlantic salmon have shown good growth performance with fishmeal incorporation at 3% or even without the finite ingredient; in such cases amino acids in the feed should be well balanced (32) and the feed should contain attractants derived from marine ingredients (31, 32). Hence, we included the essential amino acids in all the feeds. Rapeseed oil was chosen as the plant oil because it is commonly used to replace fish oil in modern aqua diets (14). The soybean meal diet (SBM; BG2) was deliberately designed to study enteritis; based on earlier reports (34–37). Soybean meal-induced inflammation model is often used to study effects of the ingredient on gut health as well as bile acid levels and hypocholesterolaemia (36–38).

This experiment was not designed as a typical growth performance trial with feed intake measurements. Nevertheless, the results showed that except for the fish fed soybean meal (BG2), all diet groups doubled their weights during the 65 days feeding trial; this result indicates that the diets generally performed well. The lower weight gain of fish fed BG2 is not an unexpected finding because previous studies have already reported such a consequence of soybean meal feeding. Fish fed BG3 diet that contains 50% fishmeal and 21% rapeseed oil had the best growth; the good growth is likely to be due to the high protein and lipid digestibility of this diet.

The lowest protein digestibility was observed for the fish fed the fishmeal and fish oil diet (BG1). Protein digestibility of fishmeal-based diet can vary between 82% to almost 90% (39, 40). However, the difference in protein digestibility between BG1 and BG3 was unexpected because both diets contained the same amount and source of fishmeal. Lipid digestibility was lower in the diets containing fish oil (BG1, BG2 and BG4). The result may be explained by the higher content of saturated fatty acids in fish oil compared to rapeseed oil (41). The lowest lipid digestibility was noted for the fish fed the SBM diet, and the finding corroborates with those of earlier studies (36–38). The highest energy digestibility was observed in fish fed fishmeal and plant oil (BG3), reflecting the high protein and lipid digestibility. Reduced DM content in feces from Atlantic salmon fed SBM or SPC is in line with other studies on salmonids (17, 36, 42, 43), and could likely to be an effect of altered expression of genes encoding aquaporins, ion transporters, tight junction and adherence junction proteins (17), leading to a loss of junction barrier integrity.

The morphological changes observed in the distal intestine of the fish fed the SBM diet were consistent with soybean meal-induced enteritis and in line with several other studies that employed 20% SBM in diets for salmonids. For the other diet groups, there were no severe signs of enteritis. Saponin is the antinutritional factor responsible for inducing enteritis in soybean meal fed Atlantic salmon (44), but severity is potentiated by other bioactive components of the plant ingredients (34). Soy protein concentrate is devoid of saponins (45) and incorporation up to 45% into marine based diets do not cause severe gut inflammatory and immune responses in Atlantic salmon (46, 47). Fish fed the fishmeal-based diets (BG1 and BG3) had normal distal intestine features and the only dietary difference between these two groups was the inclusion of rapeseed oil (BG3). The reduction of supranuclear vacuoles noted for fish fed BG4 and BG5 compared to BG1 indicated subtle plant-based diet-induced aberrations as reported in other studies. Loss of absorptive vacuoles was also reported by Katerina et al. (48); they evaluated the effect of replacement of fish oil with the alga *Schizochytrium limacinum* throughout the entire life cycle of Atlantic salmon by feeding the fish with diets low in marine ingredients. The final grow-out diets in the latter experiment contained either 10% fishmeal and 4.3% fish, or 10% fishmeal and 6.25% alga. Irrespective of the diet, the authors observed abnormal histomorphology in the distal intestine of the fish, characterized by enterocytes lacking vacuoles, abnormally tall folds with extensively developed branches and infiltration of inflammatory cells into the connective tissue. Taken together these two studies suggest that salmon compensate the lack of absorptive vacuoles by hypertrophy of the primary and secondary folds in the distal intestine. Based on the histology results from the present study we state that plant protein concentrates (not rapeseed oil) can also induce mild enteritis similar to the micromorphological changes that were noted in BG4 and BG5 that contained a mix of plant protein concentrates. It should be noted that all the diets in the present study were optimized to contain at least 1.7% EPA and docosahexaenoic acid (DHA) in the diets. The EPA+DHA content was also higher than the levels used by Katerina et al. (48). Other studies have pointed out the importance of fish oil to maintain a healthy barrier status and to maintain a good host disease resistance status. European seabass fed low levels of fish oil was not able to resist the invasive pathogens; an infection with *Vibrio anguillarum* resulted in increased translocation of the bacteria and increased fish mortality (49).

The lower cholesterol level in the fish fed the SBM diet is in line with other experiments that noticed hypocholesterolaemia as well as changes in the expression of genes involved in cholesterol biosynthetic pathways in fish fed soybean meal and lupin meal (36, 38, 50–52). The reduction in cholesterol level in the plasma of SBM fed fish is associated with saponins in SBM (34, 44, 51, 53). Fish fed the fishmeal and rapeseed oil diet (BG3) also had a numerically lower cholesterol level than BG1 but slightly higher than in fish fed plant protein mix and rapeseed oil (BG4). Sissener et al. (54) found a correlation between cholesterol level in the feed and its concentration in plasma, bile and whole fish. Therefore, the lower cholesterol in fish fed BG3 and BG4 can be partly explained by the lower content of cholesterol in these diets.

The mucosal mapping of the five diet groups revealed a consistent relationship with the growth data; the soybean meal diet group (BG2) had the smallest mean area, lowest volumetric density and an ensuing poor mucous cell-based barrier status compared to the other groups. Mucosal mapping results agree with more traditional analyses of gut health and with the overall growth performance. In contrast, both feeds containing a mix of plant protein concentrates (BG4 and BG5) had the largest mean mucous cell area, highest volumetric density and aberrant barrier status. These results suggest that the plant proteins cause enteritis in two ways; either by reducing (BG2) or increasing (BG4 and BG5) the mucous cell sizes and volumetric densities.

The present study focused on the secreted mucin genes that are expressed on certain mucosal tissues of salmon (4). Earlier studies have mainly investigated the expression of mucin genes in relation to stress (4), or as markers of parasite infestation (3, 55). Mucin genes are diagnostic markers of severe human diseases; e.g. airway disorders, inflammatory diseases, cancers (56–58). Tissue-specific expression of mucin genes−*muc2*-like genes in the distal intestine and *muc5*-like genes in the skin and gills−is consistent with previous research on Atlantic salmon (4). Sveen et al. (4) reported high expression of *muc5ac1* and *muc5b* in the skin and *muc5ac2* in the gills. In the present experiment only the expression of *muc5ac1* in the skin of fish fed the experimental diets differed significantly. Fish fed diets with high levels of plant protein concentrates had the highest expression of *muc5ac1*, but only the group fed diet with the highest incorporation of plant ingredients (BG5) had significantly higher *muc5ac1*. In the gills, the relative expression of *muc5ac2* was slightly higher than *muc5b* and the expression of both genes was significantly higher in fish fed soybean meal in the diet (BG2) and lowest in those fed the combination of plant protein ingredients and fish oil (BG4). Higher expression of *muc2* in the distal intestine of fish fed the marine ingredient-based diet (BG1) and down regulation in fish fed soybean meal (BG2) clearly indicate that this gene is correlated to intestinal health. The *muc2* has an anti-inflammatory and tumor suppressive role, and experiments with *muc2* knockout mice have shown abnormal goblet cells followed by development of colitis and colorectal cancer (59, 60). Mucus layer and microbiota structure are interdependent on each other, and the major and minor forms of O-glycosylated entities of *Muc2* in mice colon are known to have key roles in host-microbiota symbiosis (61). Diet is an important determinant of gut microbiota, and it is known that these microorganisms enhance the expression of e.g. *Muc2* and *Fut2* (galactoside 2-alpha-L-fucosyltransferase 2) to strengthen the mucus barrier and mucin glycan structure, thereby preventing the entry of microbes into the intestinal epithelium (62). The lower expression of *muc2* in fish fed BG3 compared to BG1 can only be explained by the different oil sources since both contained fishmeal. The difference between BG1 and BG4 could be due to the replacement of fishmeal with plant protein concentrates. The lower expression of *muc2* for fish fed the soybean meal diet (BG2) suggests that this gene may be used as a marker for severe intestinal inflammation.

Antimicrobial peptides are defense molecules that have key roles in disease prevention in fishes (63). The expression of both cathelicidins and defensins has been induced in salmonids subjected to bacterial challenge (64, 65). In the present study, *cathl1* was expressed in both the skin and distal intestine, but the expression was the highest in the skin of Atlantic salmon. The observation corroborates with that of Chang et al. (65); they also observed differential expression of the two cathelicidin genes in different tissues. After a bacterial challenge the expression of *cathl1* increased in some tissues but not in all (65). In the present experiment, the relative expression in the skin and distal intestine was affected by the feeds. As for the defensin genes, the gene *def1* was only expressed in the skin and it was unaffected by the feeds. On the other hand, *def3* was expressed in the distal intestine and was affected by the feeds. Similar to the *muc2* expression in the distal intestine, the expression of *def3* was the highest for fish fed BG1 and the lowest for fish fed BG2. Increased production of AMPs can be considered as a strategy of the fish to stimulate its immune system, and could serve as an alternate approach to reduce disease outbreaks in fish farms (2, 8).

In the present study, percentages of lymphocyte-like cells from the major hematopoietic organ (HK) of fish fed more plant ingredients (BG5) was significantly lower compared to those fed fishmeal-based diets (BG1 and BG3). The low content of fishmeal and fish oil in the BG5 diet may have influenced the counts of HK lymphocyte-like cells. In a study on European sea bass (66), the total number of circulating leucocytes in fish fed 100% fish oil diet was significantly higher than in fish fed plant oil diets. A study on mice has reported that a diet rich in fish oil promotes hematopoiesis (67); mice fed fish oil diet had significantly higher hematopoietic stem cells and hematopoietic progenitors in the spleen compared to mice fed low or high-fat diets. Increased phagocytic activity by macrophages is indicative of increased disease resistance competence (68). The significantly increased phagocytic ability and capacity of HK macrophage-like cells observed in the fish fed BG1 compared to those of other diets could be linked to increased dietary n-3 fatty acid, as reported previously (69–72).

Plant protein concentrates was used in the present experiment to reduce the effect of antinutritional factors. However, fish fed the plant protein concentrates (BG4 and BG5) in the present experiment also had altered histology and mucosal barrier status−loss of absorptive vacuoles, increased mucous cell volumetric densities and barrier status− compared to those fed the fishmeal as protein source (BG1 and BG3). There seems to be a close connection between nutritional status, modulations of the immune cell populations and their functions (73). We assume that higher *muc2* expression in the distal intestine of fish fed BG1 could contribute to the enhanced intestinal barrier protection as well as increase in immune cell counts and their function in head kidney indicating the importance of fishmeal and fish oil for the health of the fish.

In conclusion, the ADC values were within the normal ranges and the fish grew well on all diets, except the fish fed SBM. Fish fed the plant protein ingredients (BG4 and BG5) had lower DM content in the feces and had mild enteritis. Decreased mucous cell size and low barrier status were hallmarks of fish fed soybean meal, but increased cell size and abnormal barrier status were the features of fish fed plant protein mixes irrespective of lipid source. These results suggest two types of impact on gut health over the long term; either reduce mucosal protection or over-activate it. The four mucin genes in Atlantic salmon skin, gills, and distal intestine were affected by the ingredient composition. The expression of the antimicrobial peptide genes, *cathl1* and *def3* were also affected by the ingredients in the diets. Furthermore, higher numbers of lymphocyte-like cells, increased phagocytic ability and capacity of macrophage-like cells in head kidney as well as higher *muc2* expression in the distal intestine of fish fed the marine based diet (BG1) points to the compromised intestinal barrier in fish fed plant ingredients. These data can be combined with marker gene information to further refine dietary compositions for sustainable and acceptable healthy diets.

## Data Availability Statement

The original contributions presented in the study are included in the article/**Supplementary Material**, further inquiries can be directed to the corresponding author.

## Ethics Statement

The animal study was reviewed and approved by National Animal Research Authority (FDU: Forsøksdyrutvalget ID-5887) in Norway.

## Author Contributions

KK and MS designed the study and feed formulation. Feeding trials were supervised by MS, YG, and SS. GV helped SS in conducting the qPCR study. YP performed the cell study. TP, SØ, and KP were responsible for the stereology part of the manuscript. DD performed the histology study. SS wrote the first version of the manuscript. VK and MS edited the manuscript. All authors contributed to the article and approved the submitted version.

## Funding

This study was part of the project “*Blodanalyser av laks som metode for vurdering av tarmhelse*” funded by MABIT, project number AF0082.

## Conflict of Interest

KK is employed by BioVivo Technologies AS, Bodø, Norway. KP is employed by Quantidoc AS, Bergen, Norway.

The remaining authors declare that the research was conducted in the absence of any commercial or financial relationships that could be construed as a potential conflict of interest.

## References

[1] Kiron V. Fish immune system and its nutritional modulation for preventive health care. Anim Feed Sci Technol (2012) 173:111–33. 10.1016/j.anifeedsci.2011.12.015

[2] Masso-Silva JA Diamond G. Antimicrobial peptides from fish. Pharm (Basel) (2014) 7:265–310. 10.3390/ph7030265 PMC3978493 24594555

[3] Pérez-Sánchez J Estensoro I Redondo MJ Calduch-Giner JA Kaushik S Sitjà-Bobadilla A. Mucins as diagnostic and prognostic biomarkers in a fish-parasite model: Transcriptional and functional analysis. PloS One (2013) 8:e65457. 10.1371/journal.pone.0065457 23776483 PMC3680472

[4] Sveen LR Grammes FT Ytteborg E Takle H Jørgensen SM. Genome-wide analysis of Atlantic salmon (*Salmo salar*) mucin genes and their role as biomarkers. PloS One (2017) 12:e0189103. 10.1371/journal.pone.0189103 29236729 PMC5728529

[5] Venkatakrishnan V Padra JT Sundh H Sundell K Jin C Langeland M. Exploring the Arctic charr intestinal glycome: Evidence of increased n-glycolylneuraminic acid levels and changed host–pathogen interactions in response to inflammation. J Proteome Res (2019) 18:1760–73. 10.1021/acs.jproteome.8b00973 30848132

[6] Benktander J Venkatakrishnan V Padra JT Sundh H Sundell K Murugan AVM. Effects of size and geographical origin on Atlantic salmon, *Salmo salar*, mucin *O*-glycan repertoire. Mol Cell Proteomics (2019) 18:1183–96. 10.1074/mcp.RA119.001319 PMC6553937 30923042

[7] Joo H-S Fu C-I Otto M. Bacterial strategies of resistance to antimicrobial peptides. Philos Trans R Soc Lond Ser B: Biol Sci (2016) 371:20150292. 10.1098/rstb.2015.0292 27160595 PMC4874390

[8] Kościuczuk EM Lisowski P Jarczak J Strzałkowska N Jóźwik A Horbańczuk J. Cathelicidins: family of antimicrobial peptides. A review. Mol Biol Rep (2012) 39:10957–70. 10.1007/s11033-012-1997-x PMC3487008 23065264

[9] Gomez D Sunyer JO Salinas I. The mucosal immune system of fish: The evolution of tolerating commensals while fighting pathogens. Fish Shellfish Immunol (2013) 35:1729–39. 10.1016/j.fsi.2013.09.032 PMC3963484 24099804

[10] Jutfelt F. Barrier function of the gut. In: Farrell AP, editor. Encyclopedia of fish physiology: from genome to environment. Volume 2. Amsterdam: Academic press (2011). p. 1322–31.

[11] Torrecillas S Montero D Caballero MJ Pittman KA Custódio M Campo A. Dietary mannan oligosaccharides: counteracting the side effects of soybean meal oil inclusion on European sea bass (*Dicentrarchus labrax*) gut health and skin mucosa mucus production? Front Immunol (2015) 6:397. 10.3389/fimmu.2015.00397 26300883 PMC4525062

[12] Camilleri M Lyle BJ Madsen KL Sonnenburg J Verbeke K Wu GD. Role for diet in normal gut barrier function: developing guidance within the framework of food-labeling regulations. Am J Physiol Gastrointest Liver Physiol (2019) 317:G17–39. 10.1152/ajpgi.00063.2019 PMC6689735 31125257

[13] Aas TS Ytrestøyl T Åsgård T. Utilization of feed resources in the production of Atlantic salmon (*Salmo salar*) in Norway: An update for 2016. Aquac Rep (2019) 15:100216. 10.1016/j.aqrep.2019.100216

[14] Ytrestøyl T Aas TS Åsgård T. Utilisation of feed resources in production of Atlantic salmon (*Salmo salar*) in Norway. Aquaculture (2015) 448:365–74. 10.1016/j.aquaculture.2015.06.023

[15] Bou M Berge GM Baeverfjord G Sigholt T Østbye T-K Romarheim OH. Requirements of n-3 very long-chain PUFA in Atlantic salmon (*Salmo salar* L): effects of different dietary levels of EPA and DHA on fish performance and tissue composition and integrity. Br J Nutr (2017) 117:30–47. 10.1017/S0007114516004396 28112067

[16] Moldal T Løkka G Wiik-Nielsen J Austbø L Torstensen BE Rosenlund G. Substitution of dietary fish oil with plant oils is associated with shortened mid intestinal folds in Atlantic salmon (*Salmo salar*). BMC Vet Res (2014) 10:60. 10.1186/1746-6148-10-60 24606841 PMC3973862

[17] Hu H Kortner TM Gajardo K Chikwati E Tinsley J Krogdahl Å. Intestinal fluid permeability in Atlantic salmon (*Salmo salar* L.) is affected by dietary protein source. PloS One (2016) 11:e0167515–e0167515. 10.1371/journal.pone.0167515 27907206 PMC5132168

[18] Kiron V Park Y Siriyappagouder P Dahle D Vasanth G Dias J. Intestinal transcriptome reveals soy derivatives-linked changes in Atlantic salmon. Front Immunol (2020) 11:3013. 10.3389/fimmu.2020.596514. In press. PMC7759687 33362778

[19] Ringø E Zhou Z Vecino JLG Wadsworth S Romero J Krogdahl Å. Effect of dietary components on the gut microbiota of aquatic animals. A never-ending story? Aquac Nutr (2016) 22:219–82. 10.1111/anu.12346

[20] Dang M Pittman K Sonne C Hansson S Bach L Søndergaard J. Histological mucous cell quantification and mucosal mapping reveal different aspects of mucous cell responses in gills and skin of shorthorn sculpins (*Myoxocephalus scorpius*). Fish Shellfish Immunol (2020) 100:334–44. 10.1016/j.fsi.2020.03.020 32173449

[21] Kiron V Sørensen M Huntley M Vasanth GK Gong Y Dahle D. Defatted biomass of the microalga, *Desmodesmus* sp., can replace fishmeal in the feeds for Atlantic salmon. Front Mar Sci (2016) 3:67. 10.3389/fmars.2016.00067

[22] Sørensen M Gong Y Bjarnason F Vasanth GK Dahle D Huntley M. *Nannochloropsis oceania*-derived defatted meal as an alternative to fishmeal in Atlantic salmon feeds. PloS One (2017) 12:e0179907. 10.1371/journal.pone.0179907 28704386 PMC5509142

[23] Vasanth G Kiron V Kulkarni A Dahle D Lokesh J Kitani Y. A microbial feed additive abates intestinal inflammation in Atlantic salmon. Front Immunol (2015) 6:409. 10.3389/fimmu.2015.00409 26347738 PMC4541333

[24] Gong Y Sørensen SL Dahle D Nadanasabesan N Dias J Valente LMP. Approaches to improve utilization of *Nannochloropsis oceanica* in plant-based feeds for Atlantic salmon. Aquaculture (2020) 522:735122. 10.1016/j.aquaculture.2020.735122

[25] Bancroft JD Gamble M. Theory and practice of histological techniques. 6th edn. Churchill Livingstone: Elsevier (2008).

[26] Pittman K Pittman A Karlson S Cieplinska T Sourd P Redmond K. Body site matters: an evaluation and application of a novel histological methodology on the quantification of mucous cells in the skin of Atlantic salmon, *Salmo salar* L. J Fish Dis (2013) 36:115–27. 10.1111/jfd.12002 23009125

[27] Pittman K Sourd P Ravnøy B Espeland Ø Fiksdal IU Oen T. Novel method for quantifying salmonid mucous cells. J Fish Dis (2011) 34:931–6. 10.1111/j.1365-2761.2011.01308.x 22004586

[28] Vandesompele J De Preter K Pattyn F Poppe B Van Roy N De Paepe A. Accurate normalization of real-time quantitative RT-PCR data by geometric averaging of multiple internal control genes. Genome Biol (2002) 3:research0034.0031. 10.1186/gb-2002-3-7-research0034 12184808 PMC126239

[29] Park Y Abihssira-García IS Thalmann S Wiegertjes GF Barreda DR Olsvik PA. Imaging flow cytometry protocols for examining phagocytosis of microplastics and bioparticles by immune cells of aquatic animals. Front Immunol (2020) 11:203. 10.3389/fimmu.2020.00203 32133001 PMC7039858

[30] De Santis C Crampton VO Bicskei B Tocher DR. Replacement of dietary soy- with air classified faba bean protein concentrate alters the hepatic transcriptome in Atlantic salmon (*Salmo salar*) parr. Comp Biochem Physiol D: Genomics Proteomics (2015) 16:48–58. 10.1016/j.cbd.2015.07.005 26280368

[31] Kousoulaki K Rønnestad I Rathore R Sixten HJ Campbell P Nordrum S. Physiological responses of Atlantic salmon (*Salmo salar* L.) fed very low (3%) fishmeal diets supplemented with feeding-modulating crystalline amino acid mixes as identified in krill hydrolysate. Aquaculture (2018) 486:184–96. 10.1016/j.aquaculture.2017.12.011

[32] Zhang Y Øverland M Shearer KD Sørensen M Mydland LT Storebakken T. Optimizing plant protein combinations in fish meal-free diets for rainbow trout (*Oncorhynchus mykiss*) by a mixture model. Aquaculture (2012) 360-361:25–36. 10.1016/j.aquaculture.2012.07.003

[33] Collins SA Øverland M Skrede A Drew MD. Effect of plant protein sources on growth rate in salmonids: Meta-analysis of dietary inclusion of soybean, pea and canola/rapeseed meals and protein concentrates. Aquaculture (2013) 400-401:85–100. 10.1016/j.aquaculture.2013.03.006

[34] Krogdahl Å Gajardo K Kortner TM Penn M Gu M Berge GM. Soya saponins induce enteritis in Atlantic salmon (*Salmo salar* L.). J Agric Food Chem (2015) 63:3887–902. 10.1021/jf506242t 25798699

[35] Øverland M Sørensen M Storebakken T Penn M Krogdahl Å Skrede A. Pea protein concentrate substituting fish meal or soybean meal in diets for Atlantic salmon (*Salmo salar*)—Effect on growth performance, nutrient digestibility, carcass composition, gut health, and physical feed quality. Aquaculture (2009) 288:305–11. 10.1016/j.aquaculture.2008.12.012

[36] Sørensen M Penn M El-Mowafi A Storebakken T Chunfang C Øverland M. Effect of stachyose, raffinose and soya-saponins supplementation on nutrient digestibility, digestive enzymes, gut morphology and growth performance in Atlantic salmon (*Salmo salar*, L). Aquaculture (2011) 314:145–52. 10.1016/j.aquaculture.2011.02.013

[37] Baeverfjord G Krogdahl A. Development and regression of soybean meal induced enteritis in Atlantic salmon, *Salmo salar* L., distal intestine: a comparison with the intestines of fasted fish. J Fish Dis (1996) 19:375–87. 10.1046/j.1365-2761.1996.d01-92.x

[38] Kortner TM Gu J Krogdahl Å Bakke AM. Transcriptional regulation of cholesterol and bile acid metabolism after dietary soyabean meal treatment in Atlantic salmon (*Salmo salar* L.). Br J Nutr (2013) 109:593–604. 10.1017/S0007114512002024 22647297

[39] Gong Y Bandara T Huntley M Johnson ZI Dias J Dahle D. Microalgae *Scenedesmus* sp. as a potential ingredient in low fishmeal diets for Atlantic salmon (*Salmo salar* L.). Aquaculture (2019) 501:455–64. 10.1016/j.aquaculture.2018.11.049

[40] Sørensen M Berge GM Reitan KI Ruyter B. Microalga *Phaeodactylum tricornutum* in feed for Atlantic salmon (*Salmo salar*)—Effect on nutrient digestibility, growth and utilization of feed. Aquaculture (2016) 460:116–23. 10.1016/j.aquaculture.2016.04.010

[41] Turchini G Mailer RJ. Rapeseed (canola) oil and other monounsaturated fatty acid-rich vegetable oils. In: Turchini GM Ng W-K Tocher DR, editors. Fish oil replacement and alternative lipid sources in aquaculture feeds. Florida, USA: CRC Press (2011). p. 161–208.

[42] Refstie S Korsøen ØJ Storebakken T Baeverfjord G Lein I Roem AJ. Differing nutritional responses to dietary soybean meal in rainbow trout (*Oncorhynchus mykiss*) and Atlantic salmon (*Salmo salar*). Aquaculture (2000) 190:49–63. 10.1016/S0044-8486(00)00382-3

[43] Refstie S Storebakken T Roem AJ. Feed consumption and conversion in Atlantic salmon (*Salmo salar*) fed diets with fish meal, extracted soybean meal or soybean meal with reduced content of oligosaccharides, trypsin inhibitors, lectins and soya antigens. Aquaculture (1998) 162:301–12. 10.1016/S0044-8486(98)00222-1

[44] Knudsen D Jutfelt F Sundh H Sundell K Koppe W Frøkiær H. Dietary soya saponins increase gut permeability and play a key role in the onset of soyabean-induced enteritis in Atlantic salmon (*Salmo salar* L.). Br J Nutr (2008) 100:120–9. 10.1017/S0007114507886338 18167174

[45] Ireland PA Dziedzic SZ Kearsley MW. Saponin content of soya and some commercial soya products by means of high-performance liquid chromatography of the sapogenins. J Sci Food Agric (1986) 37:694–8. 10.1002/jsfa.2740370715

[46] Król E Douglas A Tocher DR Crampton VO Speakman JR Secombes CJ. Differential responses of the gut transcriptome to plant protein diets in farmed Atlantic salmon. BMC Genomics (2016) 17:156. 10.1186/s12864-016-2473-0 26925977 PMC4772681

[47] De Santis C Ruohonen K Tocher DR Martin SAM Król E Secombes CJ. Atlantic salmon (*Salmo salar*) parr as a model to predict the optimum inclusion of air classified faba bean protein concentrate in feeds for seawater salmon. Aquaculture (2015) 444:70–8. 10.1016/j.aquaculture.2015.03.024

[48] Katerina K Berge GM Turid M Aleksei K Grete B Trine Y. Microalgal *Schizochytrium limacinum* biomass improves growth and filet quality when used long-term as a replacement for fish oil, in modern salmon diets. Front Mar Sci (2020) 7:57. 10.3389/fmars.2020.00057

[49] Torrecillas S Caballero MJ Mompel D Montero D Zamorano MJ Robaina L. Disease resistance and response against *Vibrio anguillarum* intestinal infection in European seabass (*Dicentrarchus labrax*) fed low fish meal and fish oil diets. Fish Shellfish Immunol (2017) 67:302–11. 10.1016/j.fsi.2017.06.022 28602741

[50] Geay F Ferraresso S Zambonino-Infante JL Bargelloni L Quentel C Vandeputte M. Effects of the total replacement of fish-based diet with plant-based diet on the hepatic transcriptome of two European sea bass (*Dicentrarchus labrax*) half-sibfamilies showing different growth rates with the plant-based diet. BMC Genomics (2011) 12:522. 10.1186/1471-2164-12-522 22017880 PMC3377934

[51] Gu M Kortner TM Penn M Hansen AK Krogdahl Å. Effects of dietary plant meal and soya-saponin supplementation on intestinal and hepatic lipid droplet accumulation and lipoprotein and sterol metabolism in Atlantic salmon (*Salmo salar* L.). Br J Nutr (2014) 111:432–44. 10.1017/S0007114513002717 24507758

[52] Romarheim OH Skrede A Penn M Mydland LT Krogdahl Å Storebakken T. Lipid digestibility, bile drainage and development of morphological intestinal changes in rainbow trout (*Oncorhynchus mykiss*) fed diets containing defatted soybean meal. Aquaculture (2008) 274:329–38. 10.1016/j.aquaculture.2007.11.035

[53] Chikwati EM Venold FF Penn MH Rohloff J Refstie S Guttvik A. Interaction of soyasaponins with plant ingredients in diets for Atlantic salmon, *Salmo salar* L. Br J Nutr (2012) 107:1570–90. 10.1017/S0007114511004892 21914238

[54] Sissener NH Rosenlund G Stubhaug I Liland NS. Tissue sterol composition in Atlantic salmon (*Salmo salar* L.) depends on the dietary cholesterol content and on the dietary phytosterol:cholesterol ratio, but not on the dietary phytosterol content. Br J Nutr (2018) 119:599–609. 10.1017/S0007114517003853 29397797

[55] Marcos-López M Calduch-Giner JA Mirimin L MacCarthy E Rodger HD O’Connor I. Gene expression analysis of Atlantic salmon gills reveals mucin 5 and interleukin 4/13 as key molecules during amoebic gill disease. Sci Rep (2018) 8:13689. 10.1038/s41598-018-32019-8 30209326 PMC6135806

[56] Janssen WJ Stefanski AL Bochner BS Evans CM. Control of lung defence by mucins and macrophages: ancient defence mechanisms with modern functions. Eur Respir J (2016) 48:1201–14. 10.1183/13993003.00120-2015 PMC5120543 27587549

[57] Sheng YH Hasnain SZ Florin THJ McGuckin MA. Mucins in inflammatory bowel diseases and colorectal cancer. J Gastroenterol Hepatol (2012) 27:28–38. 10.1111/j.1440-1746.2011.06909.x 21913981

[58] Yonezawa S Higashi M Yamada N Yokoyama S Kitamoto S Kitajima S. Mucins in human neoplasms: Clinical pathology, gene expression and diagnostic application. Pathol Int (2011) 61:697–716. 10.1111/j.1440-1827.2011.02734.x 22126377

[59] Velcich A Yang W Heyer J Fragale A Nicholas C Viani S. Colorectal cancer in mice genetically deficient in the mucin Muc2. Science (2002) 295:1726–9. 10.1126/science.1069094 11872843

[60] Van der Sluis M De Koning BA De Bruijn AC Velcich A Meijerink JP Van Goudoever JB. Muc2-deficient mice spontaneously develop colitis, indicating that MUC2 is critical for colonic protection. Gastroenterology (2006) 131:117–29. 10.1053/j.gastro.2006.04.020 16831596

[61] Bergstrom K Shan X Casero D Batushansky A Lagishetty V Jacobs JP. Proximal colon–derived O-glycosylated mucus encapsulates and modulates the microbiota. Science (2020) 370:467–72. 10.1126/science.aay7367 PMC8132455 33093110

[62] Schroeder BO. Fight them or feed them: how the intestinal mucus layer manages the gut microbiota. Gastroenterol Rep (2019) 7:3–12. 10.1093/gastro/goy052 PMC6375348 30792861

[63] van der Marel M Adamek M Gonzalez SF Frost P Rombout JHWM Wiegertjes GF. Molecular cloning and expression of two β-defensin and two mucin genes in common carp (*Cyprinus carpio* L.) and their up-regulation after β-glucan feeding. Fish Shellfish Immunol (2012) 32:494–501. 10.1016/j.fsi.2011.12.008 22227003

[64] Casadei E Wang T Zou J González Vecino JL Wadsworth S Secombes CJ. Characterization of three novel β-defensin antimicrobial peptides in rainbow trout (*Oncorhynchus mykiss*). Mol Immunol (2009) 46:3358–66. 10.1016/j.molimm.2009.07.018 19709750

[65] Chang C-I Zhang Y-A Zou J Nie P Secombes CJ. Two cathelicidin genes are present in both rainbow trout (*Oncorhynchus mykiss*) and Atlantic salmon (*Salmo salar*). Antimicrob Agents Chemother (2006) 50:185–95. 10.1128/aac.50.1.185-195.2006 PMC1346769 16377685

[66] Mourente G Good JE. Bell JG. Partial substitution of fish oil with rapeseed, linseed and olive oils in diets for European sea bass (*Dicentrarchus labrax* L.): effects on flesh fatty acid composition, plasma prostaglandins E2 and F2α, immune function and effectiveness of a fish oil finishing diet. Aquac Nutr (2005) 11:25–40. 10.1111/j.1365-2095.2004.00320.x

[67] Xia S Li X-p Cheng L Han M-t Zhang M-m Shao Q-x. Fish oil-rich diet promotes hematopoiesis and alters hematopoietic niche. Endocrinology (2015) 156:2821–30. 10.1210/en.2015-1258 PMC4511132 26061726

[68] Magnadóttir B. Innate immunity of fish (overview). Fish Shellfish Immunol (2006) 20:137–51. 10.1016/j.fsi.2004.09.006 15950491

[69] Blazer VS. Piscine macrophage function and nutritional influences: A review. J Aquat Anim Health (1991) 3:77–86. 10.1577/1548-8667(1991)003<0077:PMFANI>2.3.CO;2

[70] Kiron V Fukuda H Takeuchi T Watanabe T. Essential fatty acid nutrition and defence mechanisms in rainbow trout *Oncorhynchus mykiss* . Comp Biochem Physiol A: Physiol (1995) 111:361–7. 10.1016/0300-9629(95)00042-6

[71] Sheldon WM Blazer VS. Influence of dietary lipid and temperature on bactericidal activity of channel catfish macrophages. J Aquat Anim Health (1991) 3:87–93. 10.1577/1548-8667(1991)003<0087:IODLAT>2.3.CO;2

[72] Thompson KD Tatner MF Henderson RJ. Effects of dietary (n-3) and (n-6) polyunsaturated fatty acid ratio on the immune response of Atlantic salmon, *Salmo salar* L. Aquac Nutr (1996) 2:21–31. 10.1111/j.1365-2095.1996.tb00004.x

[73] Alwarawrah Y Kiernan K MacIver NJ. Changes in nutritional status impact immune cell metabolism and function. Front Immunol (2018) 9:1055. 10.3389/fimmu.2018.01055 29868016 PMC5968375

