# Supplementary material for: Nutrient Digestibility, Growth, Mucosal Barrier Status, and Activity of Leucocytes From Head Kidney of Atlantic Salmon Fed Marine- or Plant-Derived Protein and Lipid Sources
Source: Front Immunol. 2021 Feb 19;11:623726. doi: 10.3389/fimmu.2020.623726 (PMC7934624; doi:10.3389/fimmu.2020.623726)
Supplement: Supplementary file 1 [file DataSheet_1.docx]

**Supplementary Table 1** Analyzed proximate composition (% as is) and amino acid composition (% as is) of the experimental feeds.

|  | **BG 1** | **BG 2** | **BG 3** | **BG 4** | **BG 5** |
| --- | --- | --- | --- | --- | --- |
| Moisture | 5.3 | 4.9 | 5.3 | 5.4 | 6.3 |
| Protein | 42.5 | 42.2 | 44.2 | 43.5 | 42.8 |
| Lipid | 29.0 | 28.6 | 27.5 | 26.0 | 26.0 |
| Ash | 11.2 | 9.45 | 11.4 | 7.18 | 7.02 |
| Energy (KJ/100 g) | 2000 | 2029 | 1966 | 2006 | 1994 |
| Amino acid |  |  |  |  |  |
| Alanine | 2.44 | 2.03 | 2.53 | 2.03 | 2.04 |
| Arginine | 2.35 | 2.33 | 2.53 | 2.33 | 2.35 |
| Aspartic acid | 3.50 | 3.43 | 3.75 | 3.51 | 3.51 |
| Glutamic acid | 6.92 | 8.03 | 7.53 | 9.04 | 9.04 |
| Glycine | 2.61 | 2.18 | 2.7 | 1.77 | 1.75 |
| Histidine | 1.01 | 1.02 | 1.08 | 1.18 | 1.17 |
| Hydroxyproline | 0.31 | 0.22 | 0.40 | 0.13 | 0.16 |
| Isoleucine | 1.66 | 1.64 | 1.73 | 1.67 | 1.66 |
| Leucine | 3.01 | 2.93 | 3.15 | 3.52 | 3.54 |
| Lysine | 2.89 | 2.85 | 3.08 | 3.08 | 3.05 |
| Phenylalanine | 1.67 | 1.79 | 1.82 | 2.11 | 2.10 |
| Proline | 2.19 | 2.47 | 2.32 | 2.96 | 2.88 |
| Serine | 1.81 | 1.91 | 1.94 | 2.06 | 2.04 |
| Threonine | 1.64 | 1.64 | 1.80 | 1.91 | 1.9 |
| Tyrosine | 1.25 | 1.35 | 1.38 | 1.50 | 1.50 |
| Valine | 1.96 | 1.86 | 2.05 | 1.87 | 1.88 |
| Tryptophan | 0.43 | 0.44 | 0.42 | 0.40 | 0.41 |
| Cysteine | 0.41 | 0.50 | 0.47 | 0.61 | 0.53 |
| Methionine | 1.37 | 1.67 | 1.65 | 1.75 | 1.68 |
| Sum EPA/DHA* | 5.9 | 5.8 | 1.7 | 5.6 | 1.7 |

BG1: Fishmeal + Fish oil diet; BG2: Soybean meal diet; BG3: Fishmeal + Plant oil diet; BG4: Plant ingredients + Fish oil diet; BG5: Plant ingredients + Plant oil diet.

*Sum EPA/DHA was calculated based on the content in the fish oil.

**Supplementary Table 2** Details of the primer sequences, amplicon size and PCR efficiency of target genes and the reference genes.

| **Gene name** | **Primer sequence** | **Amplicon size (bp)** | **PCR efficiency (%)** | **Accession numbers** |
| --- | --- | --- | --- | --- |
| **Target genes** |  |  |  | **UniProt/GenBank** |
| *muc2 (.1/2)* | GAGTGGGCTCTCAGATCCAG-F | 99 | 96.80% | XM_014184683.1 / XM_014170386.1 |
|  | GATGATGCGGACGGTAGTTT-R |  |  |  |
| *muc5ac1* | GACCTGCTCTGTGGAAGGAG-F | 120 | 96.72% | XM_014127075.1 |
|  | AGCACGGTGAATTCAGTTCC-R |  |  |  |
| *muc5ac2 (/4)* | TTTTCTCAGTTGCCGCTTTT-F | 92 | 98.90% - 99.82% | XM_014182329.1 |
|  | AGTCGGAGCCCATAAGAGGT-R |  |  |  |
| *muc5b* | ATTAAGAGCGATGTCTTCACAGC-F | 85 | 97.38% - 102.38% | XM_014175874.1 / XM_014126057.1 |
|  | AAGCACATGAGTCTCTCACACAA-R |  |  |  |
| *def1* | CATCATTTCCCTTCTCTTGCCC-F | 116 | 92.81% | LC387973.1 |
|  | AGAAACACAGCACAAGAATCCC-R |  |  |  |
| *def2* | CAGTGCAAGCTGATGACACA-F | - | - | LC387974.1 |
|  | CCTGGGCATAGCAGTACTTG-R |  |  |  |
| *def3* | AGGCATCTGCATCTCTACAC-F | 129 | 93.65% | LC387975.1 |
|  | ATTTACAGCATACATTCGGCCA-R |  |  |  |
| *def4* | GTGAACATTGCAACTCTTCTAAAG-F | - | - | LC387976.1 |
|  | CATTTACATCACATGTGATGGC-R |  |  |  |
| *cathl1* | CTAGCAACAACCTGAACACTG-F | 88 | 91.76% - 99.82% | AY728057.1 |
|  | CTTCTTGTCCGAATCTTCTGCATA-R |  |  |  |
| **Reference genes** |  |  |  | **GenBank** |
| *ef1ab* | TGCCCCTCCAGGATGTCTAC-F  CACGGCCCACAGGTACTG-R | 59 | 97.86% -100.13% | BG933853 |
| *rpl13* | CGCTCCAAGCTCATCCTCTTCCC-F  CCATCTTGAGTTCCTCCTCAGTGC-R | 79 | 93.32% - 100.14% | BT048949.1 |
| *rps29* | GGGTCATCAGCAGCTCTATTGG-F  AGTCCAGCTTAACAAAGCCGATG-R | 167 | 95.68% - 99.10% | BT043522.1 |
| *ubiq* | AGCTGGCCCAGAAGTACAACTGTG-F  CCACAAAAAGCACCAAGCCAAC-R | 162 | 92.99% - 95.40% | AB036060.1 |
